# Estimated Pulse Wave Velocity and All-Cause Mortality: Findings From the Health and Retirement Study

**DOI:** 10.1093/geroni/igac056

**Published:** 2022-08-23

**Authors:** Kevin S Heffernan, Janet M Wilmoth, Andrew S London

**Affiliations:** Department of Exercise Science, Falk College of Sport and Human Dynamics, Syracuse University, Syracuse, New York, USA; The Aging Studies Institute, Syracuse University, Syracuse, New York, USA; The Aging Studies Institute, Syracuse University, Syracuse, New York, USA; Department of Sociology, Maxwell School of Citizen and Public Affairs, Syracuse University, Syracuse, New York, USA; The Aging Studies Institute, Syracuse University, Syracuse, New York, USA; Department of Sociology, Maxwell School of Citizen and Public Affairs, Syracuse University, Syracuse, New York, USA

**Keywords:** Cardiovascular disease, Estimated pulse wave velocity, Mortality, Vascular aging, Vascular stiffness

## Abstract

**Background and Objectives:**

The gold standard method for the assessment of vascular aging is carotid–femoral pulse wave velocity (cfPWV). cfPWV can be estimated from 2 commonly assessed clinical variables—age and blood pressure. This analysis uses data from the Health and Retirement Study to examine the relationship between estimated pulse wave velocity (ePWV) and mortality among 9,293 middle age and older adults.

**Research Design and Methods:**

Cox proportional hazard models were used to predict mortality occurring over a 10- to 12-year period. Controls were included for sociodemographic characteristics (age, gender, race, ethnicity, wealth, income, and education), health status (history of cardiovascular disease [CVD], diabetes, and stroke and related medication use), health behaviors (smoking, physical activity, and body mass index), and CVD-related biomarkers (systolic and diastolic blood pressure, C-reactive protein, cystatin c, hemoglobin A1c, total cholesterol, and high-density lipoprotein cholesterol).

**Results:**

By 2018, 26.19% of the weighted analytic sample were reported as deceased. In the fully specified models that control for age, age-squared, systolic and diastolic blood pressure, sociodemographic variables, health status and behaviors, and biomarkers, ePWV was associated with a greater likelihood of mortality.

**Discussion and Implications:**

An estimate of PWV derived from age and blood pressure is independently associated with an increased likelihood of death in a representative sample of middle age and older adults in the United States.

**Translational Significance:** This study demonstrates an association between an estimate of vascular aging derived from chronological age and blood pressure (estimated pulse wave velocity [ePWV]) and all-cause mortality in 9,293 U.S. older adults in the Health and Retirement Study. As gold standard measures of vascular aging require in-person data collection with expensive equipment and high operator technical proficiency, ePWV holds promise as an easily obtained estimate of vascular aging that can be integrated into large cohort studies that measure age and blood pressure.

From 2014 to the onset of the coronavirus disease 2019 (COVID-19) pandemic, U.S. life expectancy declined as a result of an increase in all-cause mortality concentrated in midlife (Woolf & Schoomaker, 2019). Some of the observed midlife mortality increase was due to increases in drug overdoses and suicides, but a large component was related to increases in the midlife onset of chronic diseases that affect a range of bodily organ systems (Woolf & Schoomaker, 2019). Heart disease remains the most prominent cause of morbidity and mortality in the United States and globally (Virani et al., 2021). However, from 2010 to the beginning of the COVID-19 pandemic, heart disease mortality among middle-aged U.S. adults declined, while mortality from hypertensive diseases, pulmonary diseases, stroke, diabetes, and Alzheimer’s disease and related dementias increased (Woolf & Schoomaker, 2019). A similar pattern was also observed among older adults (Crimmins et al., 2019). Notably, each of the midlife causes of death that has recently increased in predominance is associated with accelerated vascular aging.

The stiffening of arteries is considered a hallmark of human aging in context. Even in the absence of overt heart disease, as people age, their large central arteries, such as the aorta, lose elasticity and stiffen (i.e., vascular aging). Vascular aging is a dynamic process that may, at the individual level, occur at a different rate than chronological aging (Cunha et al., 2017; Weber et al., 2020). Variation in vascular aging occurs in relation to biological, behavioral, and genetic/epigenetic factors interacting with social and environmental stressors experienced across the life course. Because it arises from processes of cumulative inequality (Ferraro & Shippee, 2009), early vascular aging as a reflection of the vascular allostatic load has emerged as an important health construct (Vlachopoulos et al., 2010). Individuals whose vascular age exceeds their chronological age are at greater risk for cardiovascular disease (CVD) and premature mortality (Bruno et al., 2020). Arterial stiffness has also been shown to precede and predict the development of hypertension (Koivistoinen et al., 2018), pulmonary disease (Roeder et al., 2020), stroke (van Sloten Thomas et al., 2015), diabetes (Muhammad et al., 2017), and Alzheimer’s disease and related dementias (Pase Matthew et al., 2016).

The gold standard method for the assessment of arterial stiffness is the measurement of carotid–femoral pulse wave velocity (cfPWV; Townsend et al., 2015). Although the measurement of cfPWV is well-standardized, simple, and noninvasive, various factors have limited its use in large cohort studies. Specifically, measurement requires specialized equipment and technical proficiency; the equipment and personnel needed to measure cfPWV are not available in resource-constrained environments or research studies that do not include in-person data collection with suitably trained and equipped personnel (Williams et al., 2018). Given the constraints related to measuring cfPWV, researchers have sought to develop and validate an alternate method for measuring pulse wave velocity (PWV; Greve et al., 2016).

Estimated pulse wave velocity (ePWV) is computed using equations that only require age and mean blood pressure inputs and may be useful as a measure of vascular aging in research studies. Preliminary evidence suggests that ePWV may be a reasonable proxy for cfPWV, with correlations between the two measures ranging from 0.52 to 0.67 in various studies (Greve et al., 2016; Hametner et al., 2021; Stamatelopoulos et al., 2021). Other research using data from the U.S.-based Atherosclerosis Risk in Communities (ARIC) Study report weaker correlations between cfPWV and ePWV in older Black (*r* = 0.31) and White adults (*r* = 0.36; Heffernan et al., 2022). Some research has shown that ePWV predicts CVD outcomes and all-cause mortality independent of traditional CVD risk factors, including age and blood pressure (Heffernan et al., 2020a; Vishram-Nielsen et al., 2020; Vlachopoulos et al., 2019); however, these findings may not be generalizable to Black and Hispanic adults in the general U.S. population (Heffernan et al., 2020a). Given the ease with which it can be calculated, its demonstrated correlation with the gold standard measure of pulse wave velocity, and emerging evidence of its predictive validity with respect to mortality, ePWV may be an inexpensive and easily attained measure of vascular aging that can be applied to large cohort studies where age and blood pressure are measured. Further validation of ePWV as a measure of vascular aging in large, diverse cohorts is needed in order for its utility to be properly evaluated.

To date, the small number of studies that have explored ePWV as a predictor of mortality have done so in European cohorts, and have not adequately taken gender, race, and ethnicity into account. Specifically, in some of these studies, participation has been limited to men (Jae, Heffernan, Kurl et al., 2020; Jae, Heffernan, Park et al., 2020), and the race and ethnicity of participants have not been specified (i.e., White, Caucasian, or “European” race and ethnicity has been presumed; Vishram-Nielsen et al., 2020). In this article, we examine the association between ePWV, as a measure of vascular aging, and all-cause mortality in a large, nationally representative cohort of middle age and older adults living in the United States. We estimate overall models that control statistically for gender, race, and ethnicity, and models stratified, respectively, by gender and by race and ethnicity. Multivariable analyses take into account potential confounders, including socioeconomic status; health behaviors (e.g., physical activity and smoking); comorbid disease conditions and associated medication use; and blood biomarkers that are correlated with CVD.

## Study Design

We analyze data from the Health and Retirement Study (HRS), which is a nationally representative prospective, longitudinal study of U.S. adults who are older than the age of 50. Started in 1992, the HRS was designed to explore patterns of later-life work, aging, and retirement in order to inform public policy (Crosswell et al., 2020). The HRS uses a steady-stage design that incorporates new cohorts every 6 years. Participants are surveyed every 2 years. In 2006, the HRS began conducting enhanced, face-to-face visits that include clinical measurements collected from a random one-half of the sample every 4 years. These measures include anthropometrics (e.g., height and weight); blood biomarkers (e.g., measures of cholesterol, inflammation, renal function, and blood glucose control); and blood pressure.

The HRS is approved by the institutional review board (IRB) of the University of Michigan, and all participants provide written informed consent. All of the data used in this study are deidentified, and most of the data are publicly available. The blood biomarker data are considered sensitive health data, and we were required to submit an application to use them. This study was determined to be exempt from review by the Syracuse University Office of Research and Integrity/IRB because all of the data were deidentified at the point that we had access to them.

For this study, baseline data were taken from nonproxy participants in 2006 and 2008 as the biomarker data were initially collected from half the sample during each of those years. HRS participants were excluded if there were missing data on any of the sociodemographic, health behavior, health conditions, or biomarker measures included in this analysis (*N* = 4,046). These measures are described in more detail later. In addition, participants were considered outliers and excluded if their age was 50 or younger (*N* = 349), body mass index (BMI) was <16 kg/m^2^ (*N* = 2), or systolic or diastolic blood pressure was <40 mmHg (*N* = 44). Participants who did not self-identify as White or Black (i.e., race) or as being of Hispanic ethnicity (among those reporting Black or White race) were also excluded because the sample was deemed by us to be too small to retain in the analysis (*N* = 214). Finally, participants with a zero on the survey weight variable in 2006 or 2008 were also excluded (*N* = 229). With these exclusions implemented, the analytic sample for this study includes 9,293 participants.

### All-Cause Mortality: Primary Outcome

The HRS uses multiple methods to track respondents over time, including gathering information from spouses and proxy respondents to determine vital status at each wave. According to Weir “Mortality ascertainment in the HRS is effectively complete” (Weir, 2016). The publicly available HRS cross-wave tracker file includes the month and year of each interview and of death. We used this information to construct a measure of survival in months and a censoring variable that is coded 1 for those who are known to have died by 2018 and 0 for living participants.

### Estimated Pulse Wave Velocity: Primary Independent Variable

Systolic and diastolic blood pressure is measured as the average of three consecutive readings, taken a minimum of 45 s apart, while participants are in the sitting position using an Omron HEM-780 intellisense automated blood pressure monitor with an attached Easy-Wrap ComFit arm cuff. For this study, ePWV is determined from the following equation: (Vlachopoulos et al., 2019).


$$\begin{array}{ll} 9.587\;- & \;\left(0.402\times age\right)+\left(4.560\;\times \;0.001\;\times \left(age^{2}\right)\right)-\\ & \;\left(2.621\;\times \;0.00001\;\times \left(age^{2}\right)\times \;MBP\right)+\\ & \;\left(3.176\;\times \;0.001\times age\times MBP\right)-\left(1.832\times 0.01\times MBP\right) \end{array}$$

In this equation, age and age-squared are expressed in years as continuous variables, and mean blood pressure (MBP) is calculated as follows: ([diastolic blood pressure] + [0.4 × (systolic blood pressure − diastolic blood pressure)]). MBP was calculated using a form factor of 0.4, rather than the traditional “1/3rd” approach, given that the derivation of MBP in previous ePWV equations utilized this same approach (“Determinants of pulse wave velocity in healthy people and in the presence of cardiovascular risk factors: ‘establishing normal and reference values’,” 2010). Use of 0.4 rather than 0.33 to calculate MBP is in keeping with changes in the pulse wave contour with advancing age and its relation to target organ damage (Papaioannou et al., 2016; Schultz et al., 2020).

It should be noted that more than one equation is available to estimate PWV, with the different equations derived from samples varying in CVD risk factor burden (Greve et al., 2016). Given the age of our study participants, we initially assumed that most participants had at least one CVD risk factor, and we verified this in preliminary analyses (see Table 1). Therefore, we chose an equation derived from a cohort with moderate-high CVD risk. ePWV estimated with this equation has been shown to predict survival in the Systolic Blood Pressure Intervention Trial, which includes hypertensive adults (Vlachopoulos et al., 2019), and in the National Health and Nutrition Examination Survey (NHANES), which is based on the general population of U.S. adults (Heffernan et al., 2020a). We subsequently performed sensitivity analyses using an equation derived from a European reference population (Greve et al., 2016) and also utilized in the MOnica Risk, Genetics, Archiving, and Monograph Prospective Cohort Project (Vishram-Nielsen et al., 2020) as follows:

**Table 1.** Weighted Descriptive Statistics of the Analytic Sample (*N* = 9,293)

Variable	Percent	Mean (*SD*)
Mortality	26.19	
Systolic blood pressure (mmHg)		132.61 (21.28)
Diastolic blood pressure (mmHg)		80.97 (12.35)
Age (years)		66.15 (9.71)
ePWV (m/s)		11.63 (2.29)
Gender		
Female (ref)	55.71	
Male	44.29	
Race and ethnicity		
Non-Hispanic White (ref)	85.02	
Non-Hispanic Black	8.38	
Hispanic	7.60	
Education		
<High school (ref)	16.45	
High school graduate	53.82	
Associate degree/some college	5.10	
College degree	13.92	
Graduate degree	10.71	
Income ($)		$73,450 ($114,637)
Wealth ($)		$551,942 ($1,292,953)
Physical activity		
More than once a week or daily	61.22	
Once a week	15.74	
One to three times a month	8.96	
Hardly ever or never (ref)	14.49	
Current smoker (1 = yes)	12.81	
Body mass index (kg/m^2^)		29.34 (5.68)
Heart disease (1 = yes)	55.04	
Antihypertensive medication(1 = yes)	49.25	
Diabetes (1 = yes)	18.97	
Diabetes medication (1 = yes)	15.41	
Stroke (1 = yes)	5.47	
Cholesterol medication (1 = yes)	40.14	
Total cholesterol/HDL ratio		5.82 (0.97)
Glycosylated haemoglobin (HbA1c, %)		4.01 (1.21)
Cystatin c (mg/L)		1.08 (0.48)
C-reactive protein (CRP, µg/L)		4.48 (7.99)

*Notes*: ePWV = estimated pulse wave velocity; HDL = high-density lipoprotein; *SD* = standard deviation.


$$\begin{array}{ll} 7.84 & -\left(0.33\times age\right)+\left(3.8\times 0.001\times \left(age^{2}\right)\right)\\ & -\left(1.97\times 0.00001\times \left(age^{2}\right)\times MBP\right)+\\ & \left(2.5\times 0.001\times age\times MBP\right)-\left(1.9\times 0.001\times MBP\right). \end{array}$$

Results for ePWV calculated using this alternative equation were substantively similar to those reported later (results available upon request).

### Covariates

The model we estimate includes exogenous sociodemographic variables, as well as several potentially confounding measures of socioeconomic attainment. Together, these social determinants of health anchor life-course processes of cumulative inequality that may be variably associated with health behavior, disease development, and vascular aging. The exogenous sociodemographic variables that we include are gender (male or female) and a combined measure of racial and ethnic identity (non-Hispanic White, non-Hispanic Black, and Hispanic White or Black). Socioeconomic attainment is measured with three variables: education (less than high school, high school graduate, associate degree/some college, college degree, and graduate degree); household income (in dollars); and household wealth (in dollars).

The model also includes several self-reported health behaviors and health conditions that are correlated with vascular aging and mortality. The health behaviors that are included are physical activity (daily or more than once a week, once a week, one to three times a month, and hardly ever or never) and current smoker (yes/no). Anthropometric status/body habitus is determined as BMI (kg/m^2^). The health conditions that are included are history of heart disease (ever been told by a physician you have heart problems, including heart attack, ischemic heart disease, and heart failure; yes/no); history of type 2 diabetes (yes/no); and history of stroke (yes/no). We also include associated medication use, including antihypertensive medications (yes/no), diabetes medications (yes/no), and cholesterol medications (yes/no).

In addition to the self-reported measures identified earlier, we include in the model a range of CVD-related blood biomarkers. These were measured from dried blood spots obtained via a finger prick during in-person data collection visits. The blood biomarker measures include a ratio of total cholesterol to high-density lipoprotein (HDL) cholesterol, as a measure of lipid metabolism; glycated hemoglobin A1c, as a measure of glucose control; cystatin c, as a measure of global kidney function; and C-reactive protein (CRP), as a measure of systemic low-grade inflammation. To account for assay and laboratory variability in biomarker values, HRS data are released with NHANES equivalent assay values (Crimmins et al., 2013), which we used for our analyses.

### Statistical Analysis Plan

We estimate Cox proportional hazard models predicting death between 2006/2008 and 2018 for the total analytic sample. This approach takes time to the event and censoring into account. We present results from three successive models with adjustments for additional covariates in each model. The first model includes continuous measures of systolic and diastolic blood pressure, as well as continuous measures of age and age-squared. In the second model, we add ePWV to determine the extent to which it contributes to the risk of mortality after taking into account the influences of its constituent components. In the final model, we add the sociodemographic variables, health behavior and conditions, medication use, and biomarker variables. In addition to presenting results from the fully specified model for the full analytic sample, we estimate models respectively stratified by gender and by race and ethnicity. Model 1 includes age, age-squared, systolic blood pressure, diastolic blood pressure, and ePWV. Model 2 adds the self-reported sociodemographic variables, health behaviors and conditions, and medication use. Model 3 adds CVD biomarkers (total cholesterol/HDL cholesterol ratio, HbA1c, cystatin c, and CRP). We conducted all analyses with HRS-provided weights using Stata/IC 16.0 for Windows (StataCorp, College Station, TX).

## Results

### Sample Description

Table 1 presents weighted descriptive statistics for the population represented by the analytic sample at baseline. At baseline, slightly more than half were between the ages of 65 and 79; 34% were between 51 and 64 years old, and the rest were 80 or older. The average systolic blood pressure (133 mmHg) was higher than the individual-level ideal, while the average diastolic blood pressure (81 mmHg) was near the ideal for individuals (Whelton et al., 2018). The average ePWV was 11.63 m/s, which is typical for this age range.

Considering the exogenous sociodemographic characteristics and baseline socioeconomic status, approximately 56% identified as female individuals, and more than three-quarters identified as non-Hispanic White individuals. Just over 8.3% identified as non-Hispanic Black individuals, and approximately 7.6% identified as either Hispanic White or Black individuals. The majority (nearly 54%) had graduated high school; 24% had attained a college degree or higher education. Mean income was $73,450, and mean wealth was $551,942.

In terms of health behaviors, conditions, and medication use, more than 61% exercised once per week or more, approximately 13% were current smokers, and mean BMI was 29.34 kg/m^2^, which is slightly higher than the ideal individual-level BMI of 25–27 kg/m^2^ for older adults (Launer & Harris, 1996). The majority (55%) had been diagnosed with heart disease, 19% had been diagnosed with diabetes, and almost 5.5% reported having had a stroke. Almost half of the sample was taking antihypertensive medication, approximately 15% were taking diabetes medicine, and 40% were taking cholesterol medication.

Biomarker indicators were slightly elevated but generally within the normal range for older adults (Crimmins et al., 2014). The mean total to HDL cholesterol ratio was 3.97, while the mean HbA1c was 4.01%, the mean cystatin c was 1.08 mg/L, and the mean CRP was 4.48 µg/L.

### ePWV and Mortality

Overall, by 2018, 26.19% of the study population had died. Table 2 presents the results of a set of nested Cox proportional hazard regression analyses predicting mortality. As expected in the absence of statistical controls for other factors, Model 1 indicates that higher systolic blood pressure and older age increase the likelihood of mortality, while higher diastolic blood pressure decreases the likelihood of mortality.

**Table 2.** Weighted Cox Proportional Hazard Models Predicting Mortality: 2006/2008–2018 Total Sample (*N* = 9,293)

Variable	Model 1		Model 2		Model 3	
	*b* (*SE*)	Hazard ratio	*b* (*SE*)	Hazard ratio	*b* (*SE*)	Hazard ratio
Diastolic blood pressure	−0.0118^***^ (0.0028)	0.99	−0.0539^***^ (0.0102)	0.95	−0.0299* (0.0142)	0.97
Systolic blood pressure	0.0066^***^ (0.0015)	1.01	−0.0206^**^ (0.0067)	0.98	−0.0200^+^ (0.0107)	0.98
Age	0.0763* (0.0296)	1.08	0.1284^***^ (0.0322)	1.14	0.1516^***^ (0.0406)	1.16
Age^2^	0.0001 (0.0002)	1.00	−0.0015^***^ (0.0004)	1.00	−0.0013* (0.0007)	1.00
ePWV			0.8902^***^ (0.2148)	2.44	0.6312* (0.3117)	1.88
Gender (ref = female)					0.5167^***^ (0.0474)	1.68
Race and ethnicity (ref = non-Hispanic White)						
Non-Hispanic Black					−0.0598 (0.0748)	0.94
Hispanic					−0.0891^***^ (0.0891)	0.72
Education (ref=<high school graduate)						
High school graduate					−0.0828 (0.0585)	0.92
Associate degree/some college					−0.0881 (0.1238)	0.92
College degree					−0.2640^**^ (0.0906)	0.77
Graduate degree					−0.3495^***^ (0.1061)	0.71
Income (thousands)					−0.00231^***^ (0.0000)	1.00
Wealth (hundred thousands)					−0.0001 (0.0000)	1.00
Physical activity (ref = hardly ever or never)						
More than once a week					−0.4886^***^ (0.0567)	0.61
Once a week					−0.3940^***^ (0.0714)	0.67
One to three times a month					−0.3105^***^ (0.0851)	0.73
Current smoker (1 = yes)					0.7049^***^ (0.0772)	2.02
Body mass index					−0.3996^**^ (0.1372)	0.67
Body mass index^2^					0.0111^**^ (0.0042)	1.01
Body mass index^3^					−0.0001^**^ (0.00004)	1.00
Heart disease (1 = yes)					0.3162^***^ (0.0990)	1.37
Antihypertensive medication (1 = yes)					−0.1724^+^ (0.0962)	0.84
Diabetes (1 = yes)					0.2139* (0.1093)	1.23
Diabetes medication (1 = yes)					0.1302 (0.1190)	1.14
Stroke (1 = yes)					0.3701^***^ (0.0922)	1.45
Cholesterol medication (1 = yes)					−0.0685 (0.0510)	0.93
Total cholesterol/HDL ratio					0.0062 (0.0198)	1.01
Glycosylated haemoglobin (HbA1c)					0.0441 (0.0285)	1.05
Cystatin c					0.3833^***^ (0.0289)	1.47
C-reactive protein (CRP)					0.0087^**^ (0.0028)	1.01
Log likelihood	−20,473.15^***^		−20,463.76^***^		−19,977.13^***^	

*Notes*: ePWV = estimated pulse wave velocity; HDL = high-density lipoprotein; *SE* = standard error.

^***^ *p* ≤ .001,

^**^ *p* ≤ .01,

*p ≤ .05,

^+^ *p* < .10.

Model 2 adds ePWV to Model 1. Net of age, age-squared, and the direct measures of blood pressure, ePWV has a large, positive, statistically significant association with mortality. Model 2 fits better than Model 1 (Chi-square = 18.8 with 1 d.f., *p* < .001). Diastolic blood pressure remains significant and negative in Model 2. However, with the addition of ePWV to the model, the coefficient on systolic blood pressure changes from positive to negative and is statistically significant.

Model 3 adds the exogenous sociodemographic and potentially confounding variables to Model 2. Model 3 fits better than Model 2 (Chi-square = 973.2 with 26 d.f., *p* < .001). Moreover, Model 3 fits better than a model that includes all of the covariates except ePWV (not shown; Chi-square = 9.5 with 1 d.f., *p* < .01), which indicates that ePWV makes a unique contribution to explaining the risk of mortality. The results observed in Model 2 for ePWV, age, age-squared, systolic blood pressure, and diastolic blood pressure remain relatively unchanged, while these other variables have independent associations with mortality in the expected directions. Mortality is significantly higher among males, current smokers, and persons with: heart disease, diabetes, stroke, higher cystatin c, and higher CRP. Mortality is significantly lower among persons who: identify as Hispanic; have a college/graduate degree; have higher income; and exercise more than once a week. BMI has a nonlinear association with mortality, which can be characterized as an inverted J-shaped curve.

### ePWV and Mortality: Stratified Analyses

In preliminary analyses using the fully specified Model 3, we tested interactions between gender and ePWV and between race/ethnicity and ePWV, respectively (results available upon request). While there was no statistically significant gender interaction, the interaction between non-Hispanic Black and ePWV was statistically significant and anomalous. Specifically, among non-Hispanic Blacks, at higher levels of ePWV, the risk of mortality was attenuated. To expand upon these preliminary analyses, Table 3 presents results from two distinct sets of supplemental analyses that explore the association of ePWV and mortality in subpopulations defined by gender and race/ethnicity, respectively. The first stratifies the sample by gender and presents results from three models separately for men and women. The second stratifies the sample by race and ethnicity and presents results separately for non-Hispanic White individuals, non-Hispanic Black individuals, and Hispanic White or Black individuals.

**Table 3.** Weighted Cox Proportional Hazard Models Predicting Mortality: 2006/2008–2018, by Gender and by Race and Ethnicity

Variable	Gender^a^				Race and ethnicity^a^					
	Men (*n* = 3,830)		Women (*n* = 5.463)		Non-Hispanic White (*n* = 7,287)		Non-Hispanic Black (*n* = 1,180)		Hispanic (*n* = 826)	
	*b* (*SE*)	Hazard ratio	*b* (*SE*)	Hazard ratio	*b* (*SE*)	Hazard ratio	*b* (*SE*)	Hazard ratio	*b* (*SE*)	Hazard ratio
Model 1^b^										
ePWV	0.9538^**^ (0.3561)	2.60	0.5731* (0.2592)	1.77	0.9784^***^ (0.2481)	2.66	0.1048 (0.4979)	1.11	0.1588 (0.8059)	1.17
Model 2^c^										
ePWV	0.8346^**^ (0.3347)	2.30	0.5966^+^ (0.3457)	1.82	0.8406^**^ (0.3159)	2.32	−0.1949 (0.5257)	0.82	−0.4210 (0.8927)	0.66
Model 3^d^										
ePWV	0.6393^+^ (0.3330)	1.90	0.5168 (0.3599)	1.68	0.7493* (0.3337)	2.12	−0.5772 (0.5316)	0.56	−0.4150 (0.8990)	0.66

*Notes*: ePWV = estimated pulse wave velocity; *SE* = standard error.

^a^The gender models include a control for race and ethnicity, while the race and ethnicity models include a control for gender.

^b^Model 1 includes diastolic blood pressure, systolic blood pressure, age and age-squared, but it does not include any controls.

^c^Model 2 adds to Model 1 controls for socioecomomic status; health behaviors (e.g., physical activity and smoking); comorbid disease conditions and medication use.

^d^Model 3 adds to Model 2 blood biomarkers that are correlated with cardiovascular disease.

^***^ *p* ≤ .001,

^**^ *p* ≤ .01,

*p ≤ .05,

^+^ *p* < .10.

The results indicate ePWV has a strong, positive, statistically significant association with mortality among both men and women following adjustment for ePWV’s constituent components—age, age-squared, and blood pressure. Among men, controlling for socioeconomic status, health behaviors (e.g., physical activity and smoking), comorbid disease conditions, and medication use in Model 2 reduce the association between ePWV and mortality by 12.5%, but the association remains statistically significant. By contrast, among women, the coefficient in Model 2 is slightly larger but relatively unchanged, the standard error increases, and the association becomes marginally significant (*p* < .10). Addition of the CVD biomarkers in Model 3 further attenuates the association of ePWV with mortality among men by 23.4% and reduces it to marginal statistical significance (*p* < .10). Among women, the association becomes nonsignificant due to a slight decrease in the size of the coefficient and a further increase in the standard error.

Considering the race/ethnicity-stratified models next, the association between ePWV and mortality remains statistically significant in all three models among non-Hispanic Whites, although from Model 1 to Model 3, the size of the coefficient is reduced by 23.4%. In Model 3, ePWV is associated with more than a twofold increase in mortality among older non-Hispanic White adults net of all of the other variables included in the model. By contrast, ePWV is not associated with mortality among non-Hispanic Black or Hispanic older adults in any of the models.

## Discussion

In this prospective, national cohort study of 9,293 U.S. middle age and older adults, we found that ePWV is associated with an increased risk of mortality over a 10- to 12-year period. Our results indicate that ePWV remains significant when adjusting statistically for the constituent components of ePWV—age, age-squared, and systolic and diastolic blood pressure. This is important in order to demonstrate that ePWV is not simply measuring the influences of age and blood pressure on mortality. Moreover, the fully specified model that includes all covariates and ePWV fits the data better than the model that includes all covariates but excludes ePWV. Our results also demonstrate that the association observed in the full sample is observed respectively among women and men (Table 3, Model 1), although adjusting for CVD risk factors and biomarkers reduces the association to marginal statistical significance among men and nonsignificance among women. Finally, among subpopulations of individuals defined in relation to race and ethnicity (i.e., non-Hispanic White individuals, non-Hispanic Black individuals, and Hispanic White or Black individuals), ePWV is associated with increased mortality only among non-Hispanic White adults. Taken together, our findings suggest that ePWV may be useful as a measure of vascular aging in research contexts where in-person biomarker data collection is possible, but the collection of the gold standard measure—cfPWV—is not. While our overall results, controlling statistically for gender and race/ethnicity, suggest that ePWV can be used in diverse samples, the additional analyses we conducted suggest that ePWV as currently computed does not perform as expected among non-Hispanic Black adults and Hispanic adults is not associated with mortality among Hispanics.

Our findings support an emerging literature that documents an independent association between ePWV and the risk of all-cause mortality (Heffernan et al., 2020a, 2020b; Vlachopoulos et al., 2019). In the current study, ePWV remained significantly associated with mortality in our fully specified model, which included such predictors of mortality as socioeconomic status (i.e., wealth, income, and education; Feinglass et al., 2007), physical activity (Wen et al., 2014), CRP (Li, Zhong et al., 2019), glycated hemoglobin (Li, Zhang et al., 2019), and blood pressure (Kim & Crimmins, 2020). As a reflection of vascular aging, loss of arterial elasticity (i.e., increasing arterial stiffness) exposes target organs to increases in pulsatile hemodynamic forces (Chirinos et al., 2019). Subsequent barotrauma causes target organ damage, which has been suggested as a potential mediator of increased mortality risk (Chirinos et al., 2019). Thus, given the other factors included in the model, we contend that our results suggest that ePWV is a proxy measure of vascular aging that is independently associated with all-cause mortality in middle age and older adults.

Our study advances the literature by including women as well as men. CVD remains the most prominent cause of mortality among women in the United States, with older women being at greater CVD risk than age-matched men (Rodgers et al., 2019). Because all female participants included in our analysis were older than the age of 50 and likely peri- and postmenopausal, the vascular-protective effects of endogenous reproductive hormones (i.e., estrogen) are largely absent. This is important to underscore as women tend to have steeper increases in arterial stiffness compared to men across the life span (Lu et al., 2020), with prominent increases in vascular aging occurring during and after the menopausal transition (DuPont et al., 2019). Moreover, women may be more susceptible to the target organ consequences of accelerated vascular aging, including a greater risk of heart failure and cognitive decline (Coutinho, 2014; Safar et al., 2020). Our gender-stratified results suggest that the association between ePWV and mortality is slightly lower among women than among men (albeit there is no statistically significant gender interaction). Similar to what is seen in men, ePWV is associated with mortality in women above and beyond its constituent components—age, age-squared, and systolic and diastolic blood pressure. Moreover, adjusting for CVD risk factors and CVD biomarkers attenuates the association between ePWV and mortality among men and women, but may have a greater mediating effect among men. Given that all biomarkers considered in the HRS contribute to/are biomarkers for vascular aging, it is not surprising that including these biomarkers attenuates associations between ePWV and mortality. Taken together, our findings support an association between ePWV and mortality in both men and women, and further underscore that there may be gender-specific factors that contribute to differential rates of vascular aging and thus mortality risk.

Our findings also advance the literature by including non-Hispanic White, non-Hispanic Black, and Hispanic White and Black middle age and older adults, and explicitly evaluate the extent to which observed associations differ within subpopulations defined by race and ethnicity. Our findings are consistent with previous research using NHANES data, which found that ePWV was not significantly associated with all-cause mortality in non-Hispanic Black and Hispanic adults (Heffernan et al., 2020a). It is possible that the discrepancy noted by race and ethnicity is related to the smaller sample sizes and lower number of deaths observed among non-Hispanic Black and Hispanic adults relative to non-Hispanic Whites in both the NHANES and the HRS. The discrepancy may also be related to limitations of the ePWV equation, although our sensitivity analysis using a different equation yielded substantively similar results. In general, currently used methods to assess CVD risk perform poorly with non-Hispanic Black and Hispanic adults in part because they were developed and validated using predominantly non-Hispanic White samples (DeFilippis et al., 2015; Rodriguez et al., 2019). The development of ePWV is no exception, with equations to estimate cfPWV from age and blood pressure being developed from European (and almost exclusively White) cohorts. Vascular aging is accelerated in Hispanic and non-Hispanic Black individuals compared to non-Hispanic White individuals (Kucharska-Newton et al., 2019). Reasons for this are unknown, but likely reflect weathering and the complex interactions of social and economic conditions, structural racism, and cumulative inequality (Geronimus et al., 2006), which have a detrimental effect on lifestyle and access to resources that promote healthy vascular aging (Churchwell et al., 2020). Such differences in rates of vascular aging may influence the association of age and blood pressure across the life span. A more inclusive ePWV equation derived from a more diverse study population may be needed to help advance the study of vascular aging in large, diverse population-based studies such as the NHANES and the HRS.

### Implications

It is generally well accepted that chronological age is a less than perfect indicator of disease burden and overall health status in aging adults (Crimmins et al., 2021). As such, the quest for a summary measure of biological age remains of critical value to the field of Geroscience. Our results suggest that ePWV might be a useful summary measure of vascular aging as it relates to mortality risk. However, ePWV should not be used as a replacement for the gold standard measure of vascular aging—cfPWV—when it can be employed. Integrating one or more measurements of cfPWV into the HRS at the same time would greatly enhance efforts to validate ePWV as a measure of vascular aging.

### Limitations and Considerations

The strengths of this study include the use of a large, heterogeneous cohort of noninstitutionalized U.S. older adults; a relatively long follow-up period; and the availability of detailed data on sociodemographic, behavioral, and clinical covariates. Our ability to include in our models measures of lipid and glucose metabolism (total cholesterol/HDL and HbA1c, respectively), systemic inflammation (CRP), and kidney function (cystatin c) is a novel contribution of this study. However, this study also has several limitations that point to directions for future research. First, we only used the baseline levels of blood pressure to calculate ePWV even though blood pressure changes over time (Mitchell et al., 2019). Future studies that consider the time-varying nature of ePWV can build on the foundations laid in this study and will provide important insights into the dynamics of vascular aging and its consequences. Second, the inadequate sample size prevented us from examining a broader range of racial and ethnic subgroups, such as individuals who identify as Asian American and those who self-identify as mixed race. The multiracial population has increased 276% in the past decade, with Asian Americans being among the fastest growing of all racial and ethnic groups in the United States. Therefore, the exploration of ePWV as a measure of vascular aging in other racial and ethnic groups is warranted. Finally, although we adjusted for a wide range of confounders, residual confounding could still exist.

Our results support a complex relationship between blood pressure and mortality in older adults that may be modified by vascular aging. Specifically, when entering ePWV into our second model that includes its constituent elements, the coefficient on systolic blood pressure changes from positive to negative and is statistically significant, while chronological age is no longer significantly associated with mortality. In the HRS, it has been shown that low systolic and diastolic blood pressure is associated with a higher risk of mortality among older adults with a history of CVD (and likely accelerated vascular aging), and that those with low systolic blood pressure have the poorest survival (Kim & Crimmins, 2020). The joint effects of blood pressure and vascular aging on mortality warrant additional study.

This study provides evidence that an estimate of PWV derived from chronological age and blood pressure is independently associated with all-cause mortality in a representative sample of middle age and older adults in the United States. We conclude that ePWV may be useful as a measure of vascular aging in large cohort studies that include the direct measurement of blood pressure or valid measures of blood pressure linked from medical records systems or other sources.
